# Supplementary material for: Differential Analysis of Key Proteins Related to Fibrosis and Inflammation in Soluble Egg Antigen of Schistosoma mansoni at Different Infection Times
Source: Pathogens. 2023 Mar 11;12(3):441. doi: 10.3390/pathogens12030441 (PMC10054402; doi:10.3390/pathogens12030441)
Supplement: Supplementary file 1 [file pathogens-12-00441-s001.zip › rev. Table S1 V0307.docx]

**Table S1.** Totally 114 proteins were identified from the SEA samples of the 8th, 10th, and 12th infection weeks of which 76 proteins were found in the 8th week of infection, 86 proteins were found in the 10th week of infection and 57 proteins were found in the 12th week of infection.

| **Predicted Function** | **Identified Protein** | **Accession Number** | **Subcellular  Localization** | **8th** | **10th** | **12th** | **SecP^*^** |
| --- | --- | --- | --- | --- | --- | --- | --- |
| Binding | 14-3-3 epsilon | Q9U491 | Cytosol / Nucleus |  | X | X | − |
|  | 14-3-3 epsilon 2 | Q95W36 | Cytosol / Nucleus | X | X |  | − |
|  | 14-3-3 protein homolog 2 | Q26537 | Cytosol / Nucleus |  | X |  | NC |
|  | Actin | A0A3Q0KUT3 | Cytoskeleton | X | X | X | − |
|  | Actin-1 | P53470 | Cytoskeleton | X | X | X | NC |
|  | Actin-2 | P53471 | Cytoskeleton | X | X | X | NC |
|  | ADP-ribosylation factor-like protein 5B | A0A3Q0KK11 | Cytosol / Nucleus | X |  |  | NC |
|  | Albumin | Q95VB7 | Extracellular | X | X | X | SP |
|  | C2 domain-containing protein | A0A3Q0KKP8 | Cytosol / Nucleus | X |  |  | − |
|  | Cadherin domain-containing protein | A0A3Q0KR77 | Membrane associated |  |  | X | SP |
|  | Calmodulin | E9LZR8 | Cytosol / Nucleus |  | X | X | NC |
|  | Calmodulin | E9LZR7 | Cytosol / Nucleus | X | X | X | NC |
|  | FERM domain-containing protein | A0A5K4F1E6 | Cytoskeleton | X |  |  | − |
|  | Histone H2A | A0A3Q0KVK3 | Cytosol / Nucleus | X |  |  | − |
|  | Histone H2B | C1M1H8 | Cytosol / Nucleus | X | X |  | − |
|  | Histone H2B | G4V6E9 | Cytosol / Nucleus | X | X | X | − |
|  | Histone H4 | C4QBN1 | Cytosol / Nucleus | X | X |  | − |
|  | Histone H4 | Q9GSS8 | Cytosol / Nucleus | X | X | X | − |
|  | LTD domain-containing protein | A0A5K4FB57 | Mitochondria |  | X |  | − |
|  | MICOS complex subunit MIC10 | A0A146MJ03 | Mitochondria |  | X |  | NC |
|  | Nucleolar pre-ribosomal-associated protein 1 | A0A3Q0KQS6 | Cytosol / Nucleus |  | X | X | − |
|  | Putative actin | G4VLW1 | Cytoskeleton | X | X | X | − |
|  | Putative alpha-actinin | G4VBW4 | Cytoskeleton |  | X | X | − |
|  | Putative alpha-actinin | A0A3Q0KE94 | Cytoskeleton | X | X | X | − |
|  | Putative cyln2 (Cytoplasmic linker protein-115) (Clip-115) | A0A3Q0KGG5 | Cytoskeleton | X |  |  | − |
|  | Putative rab15, 13, 10, 1, 35, 5, and | A0A3Q0KV72 | Cytosol / Nucleus | X | X |  | − |
|  | Putative rab9 and | G4V6R4 | Cytosol / Nucleus |  | X |  | NC |
|  | Putative sorting nexin | G4V6X9 | Membrane associated |  | X |  | − |
|  | Rab-related GTP-binding protein | Q26554 | Cytosol / Nucleus | X | X | X | − |
|  | Spectrin alpha chain | A0A5K4F8H0 | Cytoskeleton |  | X |  | − |
|  | Tubulin alpha chain | C4Q4S5 | Cytoskeleton |  | X |  | − |
|  | Tubulin alpha chain | G4VKY4 | Cytoskeleton | X | X |  | − |
|  | Tubulin alpha chain | Q26595 | Cytoskeleton | X | X |  | − |
|  | Tubulin beta chain | C4QIC0 | Cytoskeleton | X |  |  | NC |
|  | Tubulin beta chain | G4VHK8 | Cytoskeleton | X | X |  | NC |
|  | vesicle-fusing ATPase | G4M0P7 | Cytosol / Nucleus | X | X |  | − |
|  | Vasa-like DEAD-box RNA helicase | I6SIZ7 | Cytosol / Nucleus | X |  |  | NC |
|  | Putative cell division cycle | A0A5K4F8X3 | Cytosol / Nucleus | X |  |  | NC |
|  | Cadherin | A0A5K4F4W8 | Membrane associated |  |  | X | SP |
| Enzymatic | Succinate--CoA ligase [ADP/GDP-forming] subunit alpha, mitochondrial | G4VI16 | Mitochondria | X |  |  | NC |
|  | Matrix metallopeptidase-7 (M10 family) | A0A3Q0KP46 | Extracellular |  | X |  | SP |
|  | NADH dehydrogenase [ubiquinone] flavoprotein 1, mitochondrial | A0A3Q0KC95 | Mitochondria | X | X | X | NC |
|  | Glutathione S-transferase class-mu 28 kDa isozyme | P09792 | Cytosol / Nucleus |  | X |  | − |
|  | CTP synthase | A0A5K4F3N0 | Cytosol / Nucleus |  | X |  | NC |
|  | coproporphyrinogen oxidase | A0A3Q0KRI2 | Mitochondria | X | X | X | NC |
| Metabolic process | Dihydrolipoyllysine-residue succinyltransferase component of 2-oxoglutarate dehydrogenase complex, mitochondrial | A0A5K4F4M9 | Mitochondria | X |  |  | NC |
|  | CAD protein | A0A3Q0KU51 | Cytosol / Nucleus | X | X | X | − |
|  | Glutamate dehydrogenase | G4LYZ1 | Mitochondria | X | X | X | − |
|  | Isocitrate dehydrogenase [NADP] | A0A3Q0KRX7 | Mitochondria | X |  |  | − |
|  | Isocitrate dehydrogenase [NADP] | A0A5K4EW27 | Mitochondria | X | X | X | NC |
|  | Malate dehydrogenase | G4VBJ0 | Mitochondria | X | X | X | NC |
|  | Pyruvate carboxylase | A0A3Q0KND4 | Mitochondria | X |  |  | − |
|  | Aspartate aminotransferase | A0A5K4EG06 | Membrane associated | X | X | X | NC |
|  | ornithine aminotransferase | A7UAX6 | Mitochondria | X | X |  | SP |
|  | ornithine aminotransferase | A7UAX7 | Mitochondria | X | X | X | SP |
| Other | Chorein_N domain-containing protein | A0A5K4FEM2 | Unknown |  |  | X | NC |
|  | DIX domain-containing protein | G4LX28 | Unknown | X |  |  | NC |
|  | Putative titin | A0A5K4EM68 | Cytosol / Nucleus |  | X |  | − |
|  | CUB domain-containing protein | A0A5K4F1G2 | Unknown |  |  | X | − |
|  | DUF1619 domain-containing protein | A0A5K4FEM0 | Unknown |  |  | X | SP |
|  | DUF3453 domain-containing protein | A0A5K4F907 | Unknown | X |  |  | NC |
|  | Prohibitin | A0A5K4FC32 | Membrane associated | X | X |  | SP |
|  | Prohibitin | G4VS58 | Membrane associated | X | X | X | NC |
|  | Putative sarg904 | G4V6I2 | Unknown | X | X |  | SP |
|  | Uncharacterized protein | A0A5K4F9R4 | Unknown |  | X | X | NC |
|  | Uncharacterized protein | A0A3Q0KKZ9 | Unknown | X | X | X | − |
| Oxidoreductase activity | NADH dehydrogenase [ubiquinone] iron-sulfur protein 3, mitochondrial | A0A3Q0KJ51 | Mitochondria |  | X |  | NC |
|  | Succinate dehydrogenase [ubiquinone] flavoprotein subunit, mitochondrial | G4LY63 | Mitochondria | X | X | X | − |
|  | Choline dehydrogenase | A0A5K4F0H9 | Mitochondria |  | X | X | NC |
|  | Peroxiredoxin, Prx3 | G4LXH7 | Mitochondria | X |  |  | NC |
|  | Putative aldehyde dehydrogenase | G4VN20 | Mitochondria | X | X | X | NC |
|  | Glutathione transferase | A0A5K4FAQ2 | Cytosol / Nucleus |  | X |  | − |
| Protein folding | Calreticulin | A0A3Q0KDW8 | Cytosol / Nucleus | X | X |  | SP |
|  | Endoplasmin | Q9NHY5 | Cytosol / Nucleus | X | X | X | SP |
|  | T-complex protein 1 subunit beta | G4VQK1 | Cytosol / Nucleus | X | X |  | − |
|  | T-complex protein 1 subunit epsilon | G4VF75 | Membrane associated | X | X | X | − |
|  | T-complex protein 1 subunit eta | A0A3Q0KPW4 | Cytosol / Nucleus | X |  |  | − |
|  | Peptidyl-prolyl cis-trans isomerase | G4VFQ0 | Cytosol / Nucleus | X |  |  | SP |
|  | 10 kDa heat shock protein, mitochondrial | G4VML2 | Mitochondria | X | X | X | − |
|  | Heat shock 70 kDa protein homolog | P08418 | Cytosol / Nucleus |  |  | X | − |
|  | Heat shock protein 70 | A0A5K4F1Y0 | Cytosol / Nucleus | X |  |  | − |
|  | Heat shock protein HSP60 | Q8MXA4 | Cytosol / Nucleus |  | X |  | − |
|  | Heat shock protein HSP60, putative | A0A3Q0KC41 | Cytosol / Nucleus | X | X | X | − |
|  | Putative heat shock protein | A0A3Q0KHG3 | Cytosol / Nucleus | X | X | X | − |
|  | Putative heat shock protein 70 | A0A5K4F5D3 | Cytosol / Nucleus | X | X | X | − |
|  | Putative heat shock protein 70 (Hsp70) | A0A3Q0KK62 | Cytosol / Nucleus | X |  |  | − |
|  | Putative heat shock protein 70 (Hsp70) | G4V910 | Cytosol / Nucleus | X | X | X | SP |
| Regulation of biological process | Mediator of RNA polymerase II transcription subunit 8 | G4VHF6 | Cytosol / Nucleus |  |  | X | NC |
|  | Non-specific serine/threonine protein kinase | A0A3Q0KBX5 | Cytosol / Nucleus |  | X |  | − |
|  | 60S ribosomal protein L12 | G4V9G5 | Cytosol / Nucleus | X | X | X | NC |
|  | Elongation factor 1-alpha | G4VAD2 | Membrane associated |  | X | X | − |
|  | tRNA-intron lyase | A0A5K4F0S0 | Cytosol / Nucleus |  | X | X | − |
| Signal transduction | Phosphatidylinositol-4,5-bisphosphate 3-kinase catalytic subunit alpha PI3K | A0A3Q0KRX5 | Membrane associated |  | X |  | NC |
|  | Putative adenylate kinase 1 | A0A3Q0KGQ7 | Mitochondria | X | X |  | NC |
|  | Guanine nucleotide-binding protein G(S) subunit alpha | C4QDC6 | Cytosol / Nucleus |  | X |  | − |
|  | Putative gtp-binding protein (I) alpha-2 subunit, gnai2 | G4VHF0 | Membrane associated |  | X | X | NC |
|  | Putative rap1 and | G4VE67 | Membrane associated |  | X | X | NC |
|  | Putative rho2 GTPase | G4V9A8 | Membrane associated | X |  |  | NC |
|  | Putative trimeric G-protein alpha o subunit | A0A3Q0KCR4 | Membrane associated |  | X | X | − |
|  | Ras family protein | A0A5K4F3V1 | Membrane associated |  | X |  | NC |
|  | Rho2 GTPase | Q8I8A1 | Membrane associated | X | X | X | NC |
|  | Trimeric G-protein alpha o subunit | Q8IT62 | Membrane associated | X | X | X | − |
| Transport | ATP synthase subunit alpha | G4VLJ0 | Membrane associated | X | X | X | − |
|  | ATP synthase subunit beta | A0A3Q0KEM2 | Membrane associated | X | X | X | NC |
|  | Clathrin heavy chain | A0A3Q0KQD5 | Membrane associated | X | X |  | − |
|  | Exportin-2 | A0A3Q0KLC3 | Membrane associated | X | X |  | NC |
|  | Sodium/potassium-transporting ATPase subunit alpha | A0A3Q0KLP2 | Membrane associated |  | X | X | NC |
|  | Sodium/potassium-transporting ATPase subunit alpha | G4VGA5 | Membrane associated | X | X | X | − |
|  | Sodium/potassium-transporting ATPase subunit alpha | Q95WT4 | Membrane associated | X | X | X | NC |
|  | ADP/ATP translocase | A0A3Q0KI71 | Mitochondria | X | X | X | − |
|  | Multidrug and toxin extrusion protein | A0A3Q0KMD5 | Membrane associated |  | X |  | NC |
|  | Phosphate carrier protein, mitochondrial | G4V6T6 | Mitochondria | X | X | X | NC |
|  | Putative tricarboxylate transport protein | A0A5K4EQY2 | Membrane associated | X |  |  | NC |
|  | Vesicle-fusing ATPase | G4VMW9 | Cytosol / Nucleus | X | X |  | − |

“X” represents the protein found in the specific infection time.

- The prediction by secretome P (SecP) on the likelihood of the protein being secreted via the classical (SP) or non-classical (NC), or not predicted to be secreted (−).
