# Supplementary material for: Differential Analysis of Key Proteins Related to Fibrosis and Inflammation in Soluble Egg Antigen of Schistosoma mansoni at Different Infection Times
Source: Pathogens. 2023 Mar 11;12(3):441. doi: 10.3390/pathogens12030441 (PMC10054402; doi:10.3390/pathogens12030441)
Supplement: Supplementary file 1 [file pathogens-12-00441-s001.zip › rev. Table S2 V0307.docx]

**Table S2.** SEA proteins identified in each stage of the 8th, 10th, and 12th infection weeks

| **Week of infection** | **Predicted Function** | **Identified Protein** | **Accession Number** | **Subcellular  Localization** | **SecP^*^** |
| --- | --- | --- | --- | --- | --- |
| 8th week | Binding | 14-3-3 epsilon 2 | Q95W36 | Cytosol / Nucleus | – |
|  | Binding | Actin | A0A3Q0KUT3 | Cytoskeleton | – |
|  | Binding | Actin-1 | P53470 | Cytoskeleton | NC |
|  | Binding | Actin-2 | P53471 | Cytoskeleton | NC |
|  | Binding | ADP-ribosylation factor-like protein 5B | A0A3Q0KK11 | Cytosol / Nucleus | NC |
|  | Binding | Albumin | Q95VB7 | Extracellular | SP |
|  | Binding | C2 domain-containing protein | A0A3Q0KKP8 | Cytosol / Nucleus | – |
|  | Binding | Calmodulin | E9LZR7 | Cytosol / Nucleus | NC |
|  | Binding | FERM domain-containing protein | A0A5K4F1E6 | Cytoskeleton | – |
|  | Binding | Histone H2A | A0A3Q0KVK3 | Cytosol / Nucleus | – |
|  | Binding | Histone H2B | C1M1H8 | Cytosol / Nucleus | – |
|  | Binding | Histone H2B | G4V6E9 | Cytosol / Nucleus | – |
|  | Binding | Histone H4 | C4QBN1 | Cytosol / Nucleus | – |
|  | Binding | Histone H4 | Q9GSS8 | Cytosol / Nucleus | – |
|  | Binding | Putative actin | G4VLW1 | Cytoskeleton | – |
|  | Binding | Putative alpha-actinin | A0A3Q0KE94 | Cytoskeleton | – |
|  | Binding | Putative cyln2 (Cytoplasmic linker protein-115) (Clip-115) | A0A3Q0KGG5 | Cytoskeleton | – |
|  | Binding | Putative rab15, 13, 10, 1, 35, 5, and | A0A3Q0KV72 | Cytosol / Nucleus | – |
|  | Binding | Rab-related GTP-binding protein | Q26554 | Cytosol / Nucleus | – |
|  | Binding | Tubulin alpha chain | G4VKY4 | Cytoskeleton | – |
|  | Binding | Tubulin alpha chain | Q26595 | Cytoskeleton | – |
|  | Binding | Tubulin beta chain | C4QIC0 | Cytoskeleton | NC |
|  | Binding | Tubulin beta chain | G4VHK8 | Cytoskeleton | NC |
|  | Binding | vesicle-fusing ATPase | G4M0P7 | Cytosol / Nucleus | – |
|  | Binding | Vasa-like DEAD-box RNA helicase | I6SIZ7 | Cytosol / Nucleus | NC |
|  | Binding | Putative cell division cycle | A0A5K4F8X3 | Cytosol / Nucleus | NC |
|  | Enzymatic | Succinate--CoA ligase [ADP/GDP-forming] subunit alpha, mitochondrial | G4VI16 | Mitochondria | NC |
|  | Enzymatic | NADH dehydrogenase [ubiquinone] flavoprotein 1, mitochondrial | A0A3Q0KC95 | Mitochondria | NC |
|  | Enzymatic | coproporphyrinogen oxidase | A0A3Q0KRI2 | Mitochondria | NC |
|  | Metabolic process | Dihydrolipoyllysine-residue succinyltransferase component of 2-oxoglutarate dehydrogenase complex, mitochondrial | A0A5K4F4M9 | Mitochondria | NC |
|  | Metabolic process | CAD protein | A0A3Q0KU51 | Cytosol / Nucleus | – |
|  | Metabolic process | Glutamate dehydrogenase | G4LYZ1 | Mitochondria | – |
|  | Metabolic process | Isocitrate dehydrogenase [NADP] | A0A3Q0KRX7 | Mitochondria | – |
|  | Metabolic process | Isocitrate dehydrogenase [NADP] | A0A5K4EW27 | Mitochondria | NC |
|  | Metabolic process | Malate dehydrogenase | G4VBJ0 | Mitochondria | NC |
|  | Metabolic process | Pyruvate carboxylase | A0A3Q0KND4 | Mitochondria | – |
|  | Metabolic process | Aspartate aminotransferase | A0A5K4EG06 | Membrane associated | NC |
|  | Metabolic process | ornithine aminotransferase | A7UAX6 | Mitochondria | SP |
|  | Metabolic process | ornithine aminotransferase | A7UAX7 | Mitochondria | SP |
|  | Other | DIX domain-containing protein | G4LX28 | Unknown | NC |
|  | Other | DUF3453 domain-containing protein | A0A5K4F907 | Unknown | NC |
|  | Other | Prohibitin | A0A5K4FC32 | Membrane associated | SP |
|  | Other | Prohibitin | G4VS58 | Membrane associated | NC |
|  | Other | Putative sarg904 | G4V6I2 | Unknown | SP |
|  | Other | Uncharacterized protein | A0A3Q0KKZ9 | Unknown | – |
|  | Oxidoreductase activity | Succinate dehydrogenase [ubiquinone] flavoprotein subunit, mitochondrial | G4LY63 | Mitochondria | – |
|  | Oxidoreductase activity | Peroxiredoxin, Prx3 | G4LXH7 | Mitochondria | NC |
|  | Oxidoreductase activity | Putative aldehyde dehydrogenase | G4VN20 | Mitochondria | NC |
|  | Protein folding | Calreticulin | A0A3Q0KDW8 | Cytosol / Nucleus | SP |
|  | Protein folding | Endoplasmin | Q9NHY5 | Cytosol / Nucleus | SP |
|  | Protein folding | T-complex protein 1 subunit beta | G4VQK1 | Cytosol / Nucleus | – |
|  | Protein folding | T-complex protein 1 subunit epsilon | G4VF75 | Membrane associated | – |
|  | Protein folding | T-complex protein 1 subunit eta | A0A3Q0KPW4 | Cytosol / Nucleus | – |
|  | Protein folding | Peptidyl-prolyl cis-trans isomerase | G4VFQ0 | Cytosol / Nucleus | SP |
|  | Protein folding | 10 kDa heat shock protein, mitochondrial | G4VML2 | Mitochondria | – |
|  | Protein folding | Heat shock protein 70 | A0A5K4F1Y0 | Cytosol / Nucleus | – |
|  | Protein folding | Heat shock protein HSP60, putative | A0A3Q0KC41 | Cytosol / Nucleus | – |
|  | Protein folding | Putative heat shock protein | A0A3Q0KHG3 | Cytosol / Nucleus | – |
|  | Protein folding | Putative heat shock protein 70 | A0A5K4F5D3 | Cytosol / Nucleus | – |
|  | Protein folding | Putative heat shock protein 70 (Hsp70) | A0A3Q0KK62 | Cytosol / Nucleus | – |
|  | Protein folding | Putative heat shock protein 70 (Hsp70) | G4V910 | Cytosol / Nucleus | SP |
|  | Regulation of biological process | 60S ribosomal protein L12 | G4V9G5 | Cytosol / Nucleus | NC |
|  | Signal transduction | Putative adenylate kinase 1 | A0A3Q0KGQ7 | Mitochondria | NC |
|  | Signal transduction | Putative rho2 GTPase | G4V9A8 | Membrane associated | NC |
|  | Signal transduction | Rho2 GTPase | Q8I8A1 | Membrane associated | NC |
|  | Signal transduction | Trimeric G-protein alpha o subunit | Q8IT62 | Membrane associated | – |
|  | Transport | ATP synthase subunit alpha | G4VLJ0 | Membrane associated | – |
|  | Transport | ATP synthase subunit beta | A0A3Q0KEM2 | Membrane associated | NC |
|  | Transport | Clathrin heavy chain | A0A3Q0KQD5 | Membrane associated | – |
|  | Transport | Exportin-2 | A0A3Q0KLC3 | Membrane associated | NC |
|  | Transport | Sodium/potassium-transporting ATPase subunit alpha | G4VGA5 | Membrane associated | – |
|  | Transport | Sodium/potassium-transporting ATPase subunit alpha | Q95WT4 | Membrane associated | NC |
|  | Transport | ADP/ATP translocase | A0A3Q0KI71 | Mitochondria | – |
|  | Transport | Phosphate carrier protein, mitochondrial | G4V6T6 | Mitochondria | NC |
|  | Transport | Putative tricarboxylate transport protein | A0A5K4EQY2 | Membrane associated | NC |
|  | Transport | Vesicle-fusing ATPase | G4VMW9 | Cytosol / Nucleus | – |
| 10th week | Binding | 14-3-3 epsilon | Q9U491 | Cytosol / Nucleus | – |
|  | Binding | 14-3-3 epsilon 2 | Q95W36 | Cytosol / Nucleus | – |
|  | Binding | 14-3-3 protein homolog 2 | Q26537 | Cytosol / Nucleus | NC |
|  | Binding | Actin | A0A3Q0KUT3 | Cytoskeleton | – |
|  | Binding | Actin-1 | P53470 | Cytoskeleton | NC |
|  | Binding | Actin-2 | P53471 | Cytoskeleton | NC |
|  | Binding | Albumin | Q95VB7 | Extracellular | SP |
|  | Binding | Calmodulin | E9LZR8 | Cytosol / Nucleus | NC |
|  | Binding | Calmodulin | E9LZR7 | Cytosol / Nucleus | NC |
|  | Binding | Histone H2B | C1M1H8 | Cytosol / Nucleus | – |
|  | Binding | Histone H2B | G4V6E9 | Cytosol / Nucleus | – |
|  | Binding | Histone H4 | C4QBN1 | Cytosol / Nucleus | – |
|  | Binding | Histone H4 | Q9GSS8 | Cytosol / Nucleus | – |
|  | Binding | LTD domain-containing protein | A0A5K4FB57 | Mitochondria | – |
|  | Binding | MICOS complex subunit MIC10 | A0A146MJ03 | Mitochondria | NC |
|  | Binding | Nucleolar pre-ribosomal-associated protein 1 | A0A3Q0KQS6 | Cytosol / Nucleus | – |
|  | Binding | Putative actin | G4VLW1 | Cytoskeleton | – |
|  | Binding | Putative alpha-actinin | G4VBW4 | Cytoskeleton | – |
|  | Binding | Putative alpha-actinin | A0A3Q0KE94 | Cytoskeleton | – |
|  | Binding | Putative rab15, 13, 10, 1, 35, 5, and | A0A3Q0KV72 | Cytosol / Nucleus | – |
|  | Binding | Putative rab9 and | G4V6R4 | Cytosol / Nucleus | NC |
|  | Binding | Putative sorting nexin | G4V6X9 | Membrane associated | – |
|  | Binding | Rab-related GTP-binding protein | Q26554 | Cytosol / Nucleus | – |
|  | Binding | Spectrin alpha chain | A0A5K4F8H0 | Cytoskeleton | – |
|  | Binding | Tubulin alpha chain | C4Q4S5 | Cytoskeleton | – |
|  | Binding | Tubulin alpha chain | G4VKY4 | Cytoskeleton | – |
|  | Binding | Tubulin alpha chain | Q26595 | Cytoskeleton | – |
|  | Binding | Tubulin beta chain | G4VHK8 | Cytoskeleton | NC |
|  | Binding | vesicle-fusing ATPase | G4M0P7 | Cytosol / Nucleus | – |
|  | Enzymatic | Matrix metallopeptidase-7 (M10 family) | A0A3Q0KP46 | Extracellular | SP |
|  | Enzymatic | NADH dehydrogenase [ubiquinone] flavoprotein 1, mitochondrial | A0A3Q0KC95 | Mitochondria | NC |
|  | Enzymatic | Glutathione S-transferase class-mu 28 kDa isozyme | P09792 | Cytosol / Nucleus | – |
|  | Enzymatic | CTP synthase | A0A5K4F3N0 | Cytosol / Nucleus | NC |
|  | Enzymatic | coproporphyrinogen oxidase | A0A3Q0KRI2 | Mitochondria | NC |
|  | Metabolic process | CAD protein | A0A3Q0KU51 | Cytosol / Nucleus | – |
|  | Metabolic process | Glutamate dehydrogenase | G4LYZ1 | Mitochondria | – |
|  | Metabolic process | Isocitrate dehydrogenase [NADP] | A0A5K4EW27 | Mitochondria | NC |
|  | Metabolic process | Malate dehydrogenase | G4VBJ0 | Mitochondria | NC |
|  | Metabolic process | Aspartate aminotransferase | A0A5K4EG06 | Membrane associated | NC |
|  | Metabolic process | ornithine aminotransferase | A7UAX6 | Mitochondria | SP |
|  | Metabolic process | ornithine aminotransferase | A7UAX7 | Mitochondria | SP |
|  | Other | Putative titin | A0A5K4EM68 | Cytosol / Nucleus | – |
|  | Other | Prohibitin | A0A5K4FC32 | Membrane associated | SP |
|  | Other | Prohibitin | G4VS58 | Membrane associated | NC |
|  | Other | Putative sarg904 | G4V6I2 | Unknown | SP |
|  | Other | Uncharacterized protein | A0A5K4F9R4 | Unknown | NC |
|  | Other | Uncharacterized protein | A0A3Q0KKZ9 | Unknown | – |
|  | Oxidoreductase activity | NADH dehydrogenase [ubiquinone] iron-sulfur protein 3, mitochondrial | A0A3Q0KJ51 | Mitochondria | NC |
|  | Oxidoreductase activity | Succinate dehydrogenase [ubiquinone] flavoprotein subunit, mitochondrial | G4LY63 | Mitochondria | – |
|  | Oxidoreductase activity | Choline dehydrogenase | A0A5K4F0H9 | Mitochondria | NC |
|  | Oxidoreductase activity | Putative aldehyde dehydrogenase | G4VN20 | Mitochondria | NC |
|  | Oxidoreductase activity | Glutathione transferase | A0A5K4FAQ2 | Cytosol / Nucleus | – |
|  | Protein folding | Calreticulin | A0A3Q0KDW8 | Cytosol / Nucleus | SP |
|  | Protein folding | Endoplasmin | Q9NHY5 | Cytosol / Nucleus | SP |
|  | Protein folding | T-complex protein 1 subunit beta | G4VQK1 | Cytosol / Nucleus | – |
|  | Protein folding | T-complex protein 1 subunit epsilon | G4VF75 | Membrane associated | – |
|  | Protein folding | 10 kDa heat shock protein, mitochondrial | G4VML2 | Mitochondria | – |
|  | Protein folding | Heat shock protein HSP60 | Q8MXA4 | Cytosol / Nucleus | – |
|  | Protein folding | Heat shock protein HSP60, putative | A0A3Q0KC41 | Cytosol / Nucleus | – |
|  | Protein folding | Putative heat shock protein | A0A3Q0KHG3 | Cytosol / Nucleus | – |
|  | Protein folding | Putative heat shock protein 70 | A0A5K4F5D3 | Cytosol / Nucleus | – |
|  | Protein folding | Putative heat shock protein 70 (Hsp70) | G4V910 | Cytosol / Nucleus | SP |
|  | Regulation of biological process | Non-specific serine/threonine protein kinase | A0A3Q0KBX5 | Cytosol / Nucleus | – |
|  | Regulation of biological process | 60S ribosomal protein L12 | G4V9G5 | Cytosol / Nucleus | NC |
|  | Regulation of biological process | Elongation factor 1-alpha | G4VAD2 | Membrane associated | – |
|  | Regulation of biological process | tRNA-intron lyase | A0A5K4F0S0 | Cytosol / Nucleus | – |
|  | Signal transduction | Phosphatidylinositol-4,5-bisphosphate 3-kinase catalytic subunit alpha PI3K | A0A3Q0KRX5 | Membrane associated | NC |
|  | Signal transduction | Putative adenylate kinase 1 | A0A3Q0KGQ7 | Mitochondria | NC |
|  | Signal transduction | Guanine nucleotide-binding protein G(S) subunit alpha | C4QDC6 | Cytosol / Nucleus | – |
|  | Signal transduction | Putative gtp-binding protein (I) alpha-2 subunit, gnai2 | G4VHF0 | Membrane associated | NC |
|  | Signal transduction | Putative rap1 and | G4VE67 | Membrane associated | NC |
|  | Signal transduction | Putative trimeric G-protein alpha o subunit | A0A3Q0KCR4 | Membrane associated | – |
|  | Signal transduction | Ras family protein | A0A5K4F3V1 | Membrane associated | NC |
|  | Signal transduction | Rho2 GTPase | Q8I8A1 | Membrane associated | NC |
|  | Signal transduction | Trimeric G-protein alpha o subunit | Q8IT62 | Membrane associated | – |
|  | Transport | ATP synthase subunit alpha | G4VLJ0 | Membrane associated | – |
|  | Transport | ATP synthase subunit beta | A0A3Q0KEM2 | Membrane associated | NC |
|  | Transport | Clathrin heavy chain | A0A3Q0KQD5 | Membrane associated | – |
|  | Transport | Exportin-2 | A0A3Q0KLC3 | Membrane associated | NC |
|  | Transport | Sodium/potassium-transporting ATPase subunit alpha | A0A3Q0KLP2 | Membrane associated | NC |
|  | Transport | Sodium/potassium-transporting ATPase subunit alpha | G4VGA5 | Membrane associated | – |
|  | Transport | Sodium/potassium-transporting ATPase subunit alpha | Q95WT4 | Membrane associated | NC |
|  | Transport | ADP/ATP translocase | A0A3Q0KI71 | Mitochondria | – |
|  | Transport | Multidrug and toxin extrusion protein | A0A3Q0KMD5 | Membrane associated | NC |
|  | Transport | Phosphate carrier protein, mitochondrial | G4V6T6 | Mitochondria | NC |
|  | Transport | Vesicle-fusing ATPase | G4VMW9 | Cytosol / Nucleus | – |
| 12th week | Binding | 14-3-3 epsilon | Q9U491 | Cytosol / Nucleus | – |
|  | Binding | Actin | A0A3Q0KUT3 | Cytoskeleton | – |
|  | Binding | Actin-1 | P53470 | Cytoskeleton | NC |
|  | Binding | Actin-2 | P53471 | Cytoskeleton | NC |
|  | Binding | Albumin | Q95VB7 | Extracellular | SP |
|  | Binding | Cadherin domain-containing protein | A0A3Q0KR77 | Membrane associated | SP |
|  | Binding | Calmodulin | E9LZR8 | Cytosol / Nucleus | NC |
|  | Binding | Calmodulin | E9LZR7 | Cytosol / Nucleus | NC |
|  | Binding | Histone H2B | G4V6E9 | Cytosol / Nucleus | – |
|  | Binding | Histone H4 | Q9GSS8 | Cytosol / Nucleus | – |
|  | Binding | Nucleolar pre-ribosomal-associated protein 1 | A0A3Q0KQS6 | Cytosol / Nucleus | – |
|  | Binding | Putative actin | G4VLW1 | Cytoskeleton | – |
|  | Binding | Putative alpha-actinin | G4VBW4 | Cytoskeleton | – |
|  | Binding | Putative alpha-actinin | A0A3Q0KE94 | Cytoskeleton | – |
|  | Binding | Rab-related GTP-binding protein | Q26554 | Cytosol / Nucleus | – |
|  | Binding | Cadherin | A0A5K4F4W8 | Membrane associated | SP |
|  | Enzymatic | NADH dehydrogenase [ubiquinone] flavoprotein 1, mitochondrial | A0A3Q0KC95 | Mitochondria | NC |
|  | Enzymatic | coproporphyrinogen oxidase | A0A3Q0KRI2 | Mitochondria | NC |
|  | Metabolic process | CAD protein | A0A3Q0KU51 | Cytosol / Nucleus | – |
|  | Metabolic process | Glutamate dehydrogenase | G4LYZ1 | Mitochondria | – |
|  | Metabolic process | Isocitrate dehydrogenase [NADP] | A0A5K4EW27 | Mitochondria | NC |
|  | Metabolic process | Malate dehydrogenase | G4VBJ0 | Mitochondria | NC |
|  | Metabolic process | Aspartate aminotransferase | A0A5K4EG06 | Membrane associated | NC |
|  | Metabolic process | ornithine aminotransferase | A7UAX7 | Mitochondria | SP |
|  | Other | Chorein_N domain-containing protein | A0A5K4FEM2 | Unknown | NC |
|  | Other | CUB domain-containing protein | A0A5K4F1G2 | Unknown | – |
|  | Other | DUF1619 domain-containing protein | A0A5K4FEM0 | Unknown | SP |
|  | Other | Prohibitin | G4VS58 | Membrane associated | NC |
|  | Other | Uncharacterized protein | A0A5K4F9R4 | Unknown | NC |
|  | Other | Uncharacterized protein | A0A3Q0KKZ9 | Unknown | – |
|  | Oxidoreductase activity | Succinate dehydrogenase [ubiquinone] flavoprotein subunit, mitochondrial | G4LY63 | Mitochondria | – |
|  | Oxidoreductase activity | Choline dehydrogenase | A0A5K4F0H9 | Mitochondria | NC |
|  | Oxidoreductase activity | Putative aldehyde dehydrogenase | G4VN20 | Mitochondria | NC |
|  | Protein folding | Endoplasmin | Q9NHY5 | Cytosol / Nucleus | SP |
|  | Protein folding | T-complex protein 1 subunit epsilon | G4VF75 | Membrane associated | – |
|  | Protein folding | 10 kDa heat shock protein, mitochondrial | G4VML2 | Mitochondria | – |
|  | Protein folding | Heat shock 70 kDa protein homolog | P08418 | Cytosol / Nucleus | – |
|  | Protein folding | Heat shock protein HSP60, putative | A0A3Q0KC41 | Cytosol / Nucleus | – |
|  | Protein folding | Putative heat shock protein | A0A3Q0KHG3 | Cytosol / Nucleus | – |
|  | Protein folding | Putative heat shock protein 70 | A0A5K4F5D3 | Cytosol / Nucleus | – |
|  | Protein folding | Putative heat shock protein 70 (Hsp70) | G4V910 | Cytosol / Nucleus | SP |
|  | Regulation of biological process | Mediator of RNA polymerase II transcription subunit 8 | G4VHF6 | Cytosol / Nucleus | NC |
|  | Regulation of biological process | 60S ribosomal protein L12 | G4V9G5 | Cytosol / Nucleus | NC |
|  | Regulation of biological process | Elongation factor 1-alpha | G4VAD2 | Membrane associated | – |
|  | Regulation of biological process | tRNA-intron lyase | A0A5K4F0S0 | Cytosol / Nucleus | – |
|  | Signal transduction | Putative gtp-binding protein (I) alpha-2 subunit, gnai2 | G4VHF0 | Membrane associated | NC |
|  | Signal transduction | Putative rap1 and | G4VE67 | Membrane associated | NC |
|  | Signal transduction | Putative trimeric G-protein alpha o subunit | A0A3Q0KCR4 | Membrane associated | – |
|  | Signal transduction | Rho2 GTPase | Q8I8A1 | Membrane associated | NC |
|  | Signal transduction | Trimeric G-protein alpha o subunit | Q8IT62 | Membrane associated | – |
|  | Transport | ATP synthase subunit alpha | G4VLJ0 | Membrane associated | – |
|  | Transport | ATP synthase subunit beta | A0A3Q0KEM2 | Membrane associated | NC |
|  | Transport | Sodium/potassium-transporting ATPase subunit alpha | A0A3Q0KLP2 | Membrane associated | NC |
|  | Transport | Sodium/potassium-transporting ATPase subunit alpha | G4VGA5 | Membrane associated | – |
|  | Transport | Sodium/potassium-transporting ATPase subunit alpha | Q95WT4 | Membrane associated | NC |
|  | Transport | ADP/ATP translocase | A0A3Q0KI71 | Mitochondria | – |
|  | Transport | Phosphate carrier protein, mitochondrial | G4V6T6 | Mitochondria | NC |

- The prediction by secretome P (SecP) on the likelihood of the protein being secreted via the classical (SP) or non-classical (NC), or not predicted to be secreted (−).
