# Supplementary figures and images for: Differential Analysis of Key Proteins Related to Fibrosis and Inflammation in Soluble Egg Antigen of Schistosoma mansoni at Different Infection Times
Source: Pathogens. 2023 Mar 11;12(3):441. doi: 10.3390/pathogens12030441 (PMC10054402; doi:10.3390/pathogens12030441)

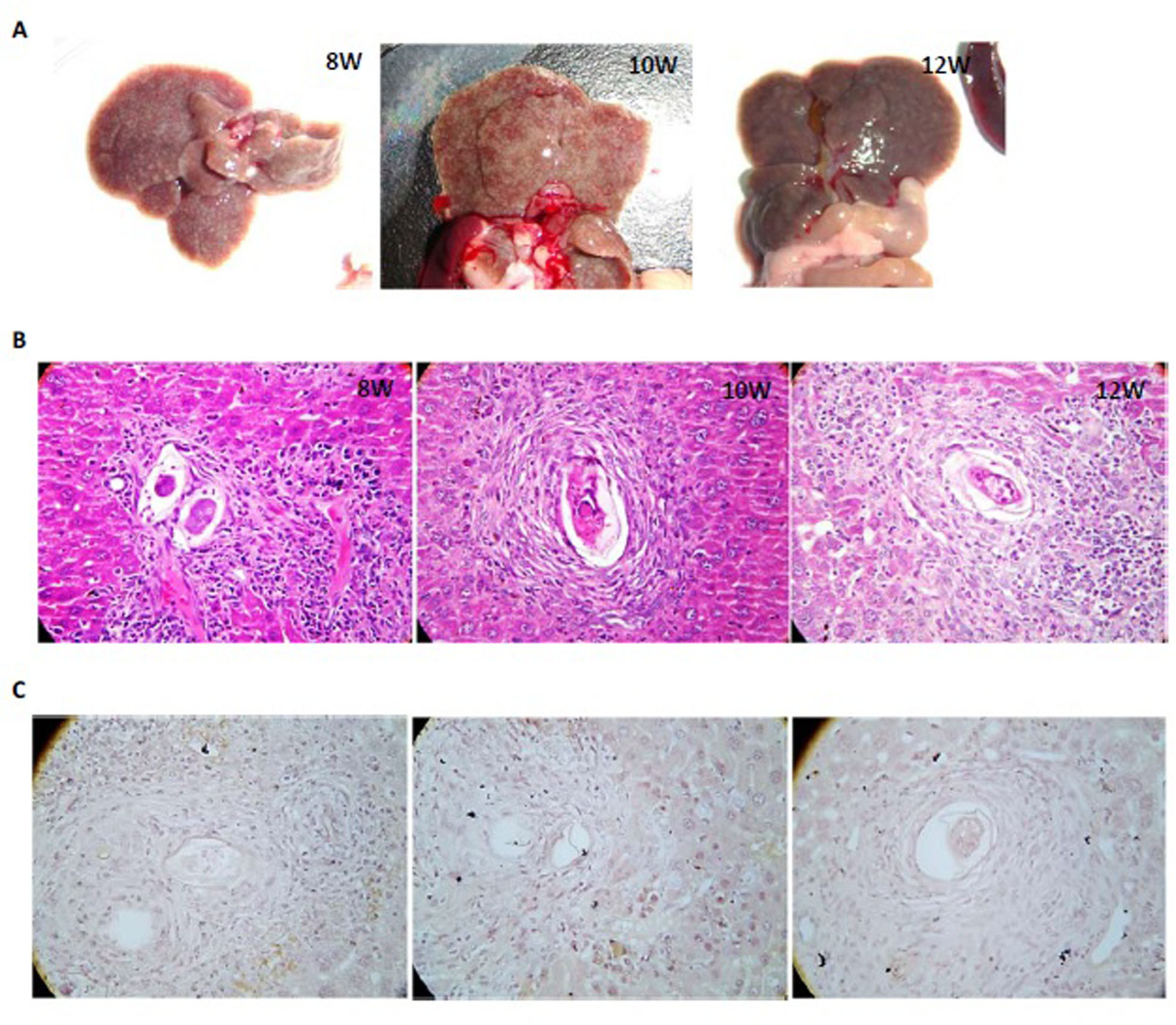

Supplement: Supplementary file 1 [file pathogens-12-00441-s001.zip › rev. Figure S1 V0307.jpg]
